# Supplementary material for: One-step formation of polymorphous sperm-like microswimmers by vortex turbulence-assisted microfluidics
Source: Nat Commun. 2024 Jun 4;15:4761. doi: 10.1038/s41467-024-49043-0 (PMC11150408; doi:10.1038/s41467-024-49043-0)
Supplement: Supplementary file 1 — Supplementary Information [file 41467_2024_49043_MOESM1_ESM.pdf]

Supplementary Information for

One-step formation of polymorphous sperm-like microswimmers by vortex turbulence assisted microfluidic

Rong Tan[1†], Xiong Yang[1†], Haojian Lu[2,3], Yajing Shen[1,4*]

[1] Department of Electronic and Computer Engineering, The Hong Kong University of Science and Technology, Clear Water Bay, Hong Kong, China

[2]State Key Laboratory of Industrial Control and Technology, Zhejiang University, Hangzhou 310027, China

[3]Institute of Cyber-Systems and Control, the Department of Control Science and Engineering, Zhejiang University, Hangzhou 310027, China

[4]Center for Smart Manufacturing, The Hong Kong University of Science and Technology, Clear Water Bay, Hong Kong, China

†These authors contributed equally to this work.

*Correspondence to: Yajing Shen (eeyajing@ust.hk)

This PDF file includes:

Figures S1-S16
Table S1-3
Note 1- Droplet formation
Note 2- Demulsification
Note 3- The description of COMSOL model setup
Note 4- Solidification
Note 5- The explanation of physical principles and simulation
Note 6- The dynamic model of sperm liked microswimmer
Note 7- The numerical calculations of propulsion
Note 8- The swelling behavior of ACA coated PSMs

Supplementary Figures

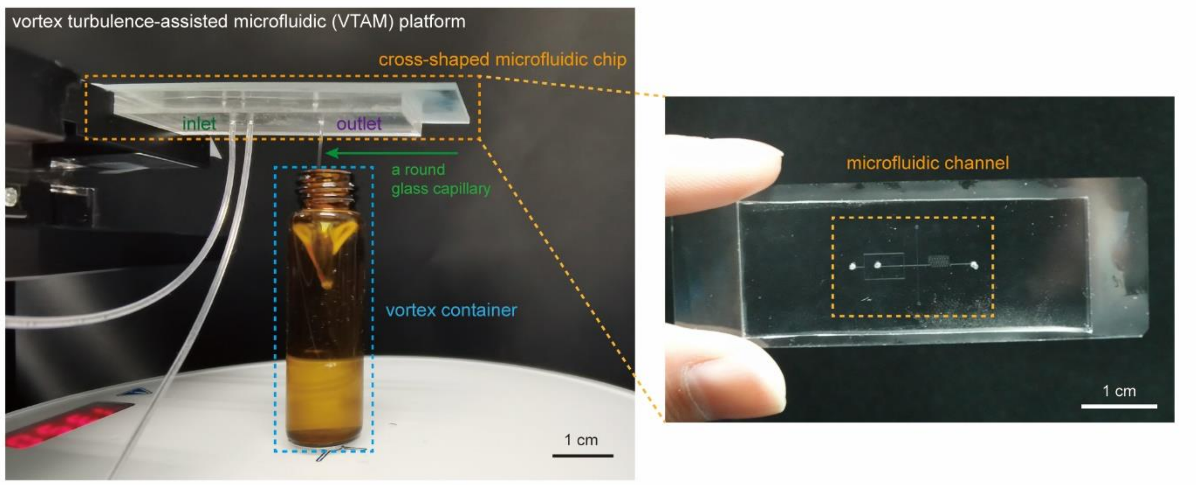

Supplementary Figure 1 | VTAM platform. Front view of the platform. The connection of the inlet with the syringe pumps. The platform consists of a cross-shaped microfluidic chip and a vortex container connected by a round glass capillary. Enlarged view of the microfluidic chip with detail designed microchannel.

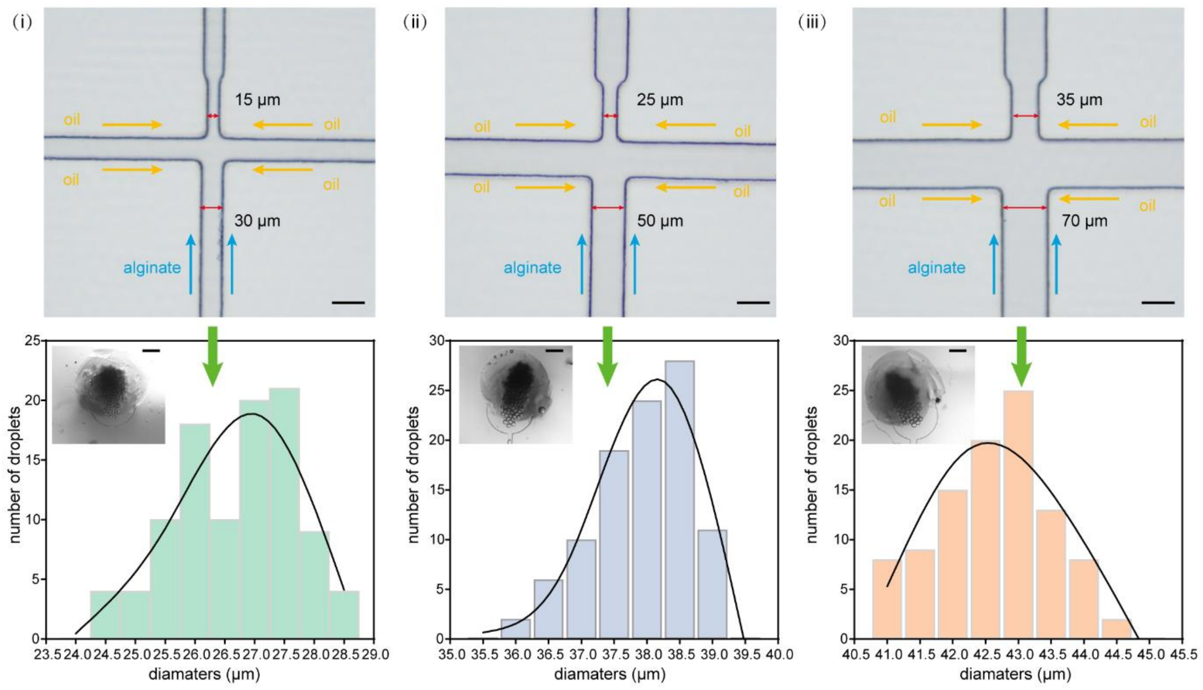

Supplementary Figure 2 | The relationships between droplet diameter and the width of channel. The width of channel (i) max: 30μm mini:15 μm (ii) max: 50μm mini:25 μm (iii) max: 70μm mini:35 μm. With the increase of channel's width, the diameter of droplet grows. The influence of channel's width on the size of desired diameters: diameter distribution of droplets with SD value (i) 27.5±0.83 μm (ii) 38.6±0.12 μm (iii) 43±0.28 μm

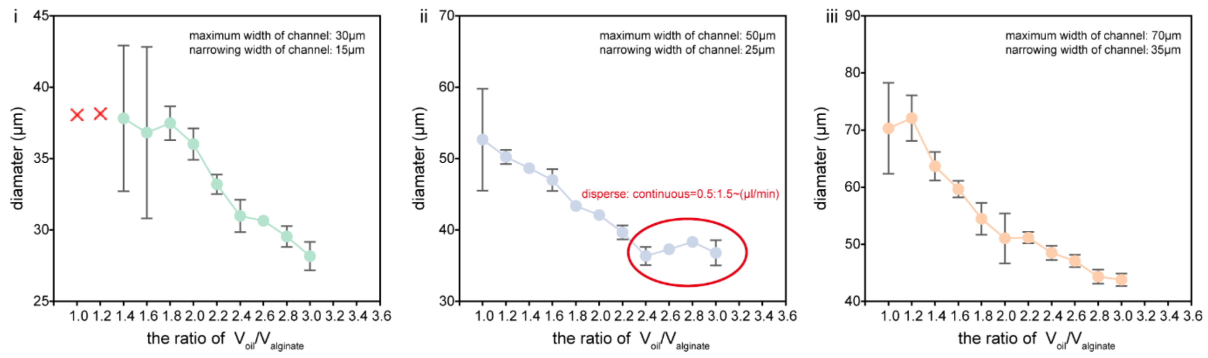

Supplementary Figure 3 | The relationships between the droplets' diameter and the two-phase flow rate ratio. Under the three widths of microfluidic channel, the influence of two-phase flow rate ratio on the size of droplet. The range of $V_{oil}/V_{alginate}$ is between from 1 to 3, when the width of channel (i) max: 30μm mini:15 μm (ii) max: 50μm mini:25 μm (iii) max: 70μm mini:35 μm. By observing the data, the acquisition of optimal droplet's size when the two-phase flow rate ratio should be set in the range of 2.4 to 3 in the 50 μm channel (ii). The curve is relatively flat in this range. Error bars indicate the standard deviation for n = 3 measurements at each data point.

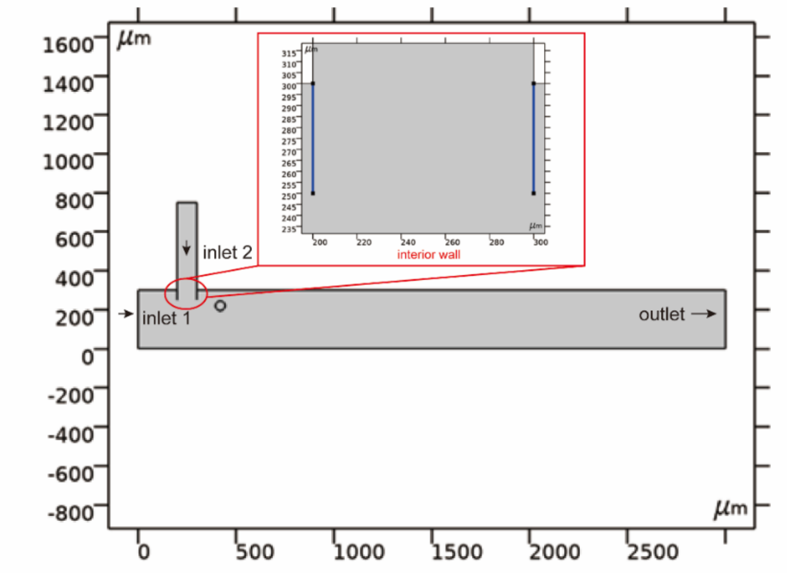

Supplementary Figure 4 | The geometry and boundaries condition of the simulation set-up.

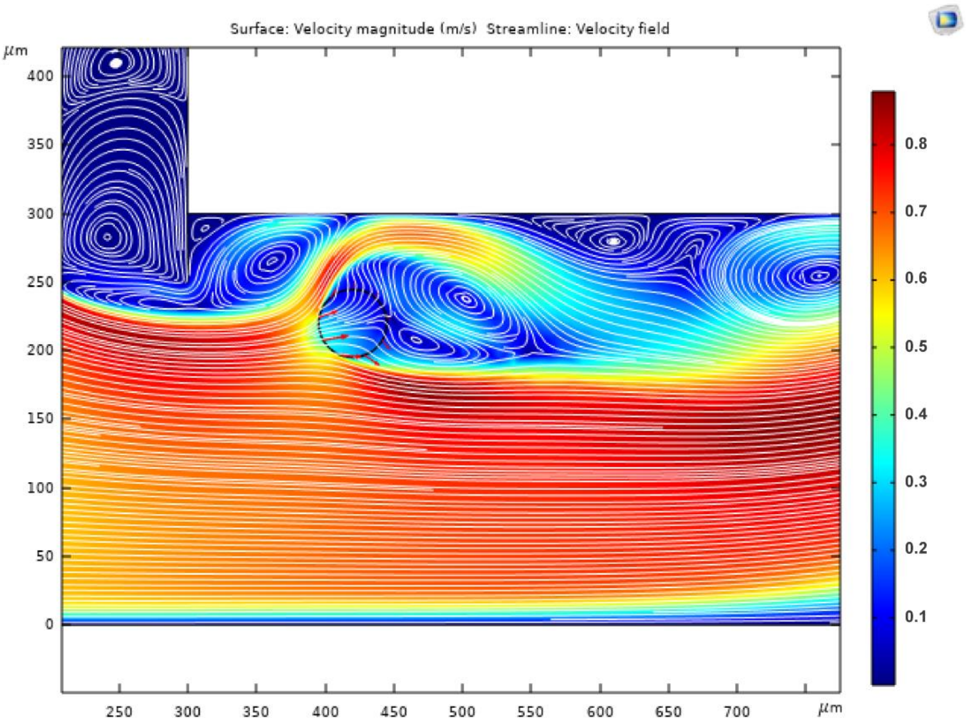

Supplementary Figure 5 | The COMSOL simulated velocity field of the droplets are in a vortex flow and are being impacted from all directions.

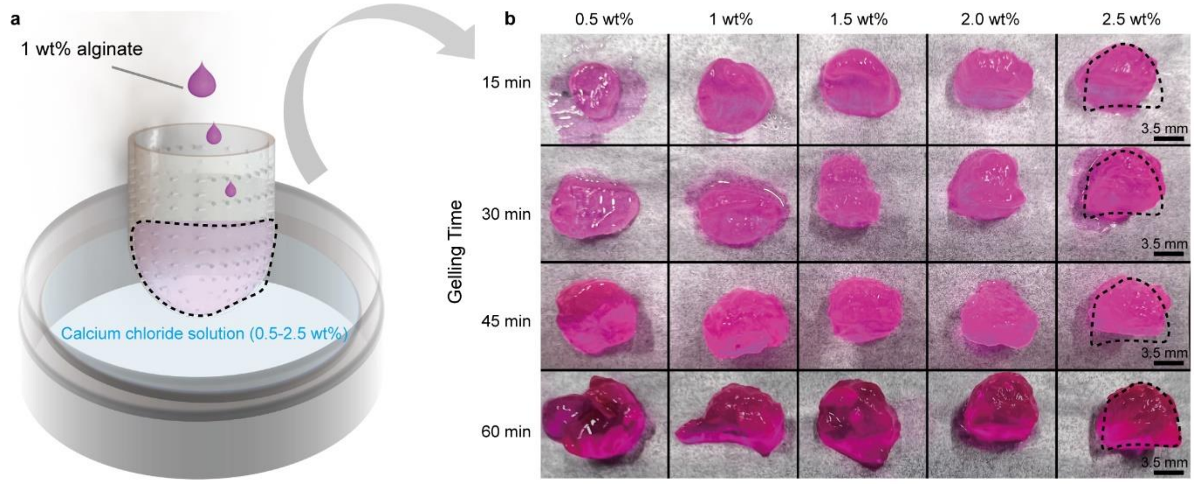

Supplementary Figure 6 | The effect of Ca^{2+} on gelling speed. a, Representative alginate gel images showing the pattern of alginate gel formation in a cap-shaped mold using external gelation method. b, The cap-shaped gel morphology is demonstrated with gelling time (15–60 min) and $CaCl_2$ concentrations (0.5-2.5 wt%).

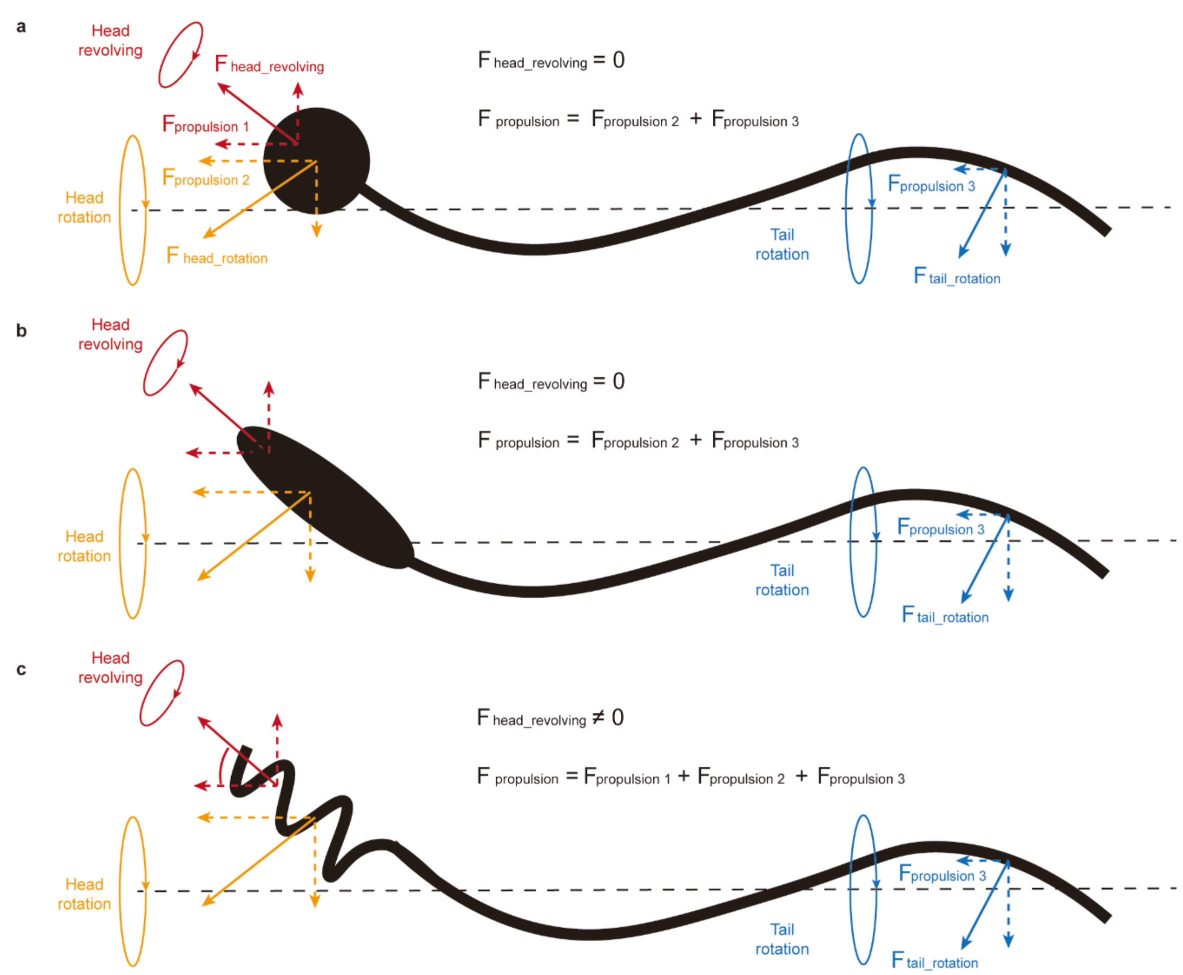

Supplementary Figure 7 | The mechanical analysis of polymorphous sperm-like microswimmers. a, The propulsion of sperm-like microswimmer with regular head including the tangential components from head rotation and tail rotation. b, Similar to the sperm-like microswimmer with regular head, only the tangential components from head rotation and tail rotation make positive work to the propulsion of sperm-like microswimmer with irregular head. c, The propulsion of sperm-like microswimmer with helix head comes from not only the tangential components of head rotation and tail rotation but also the tangential component of head revolving.

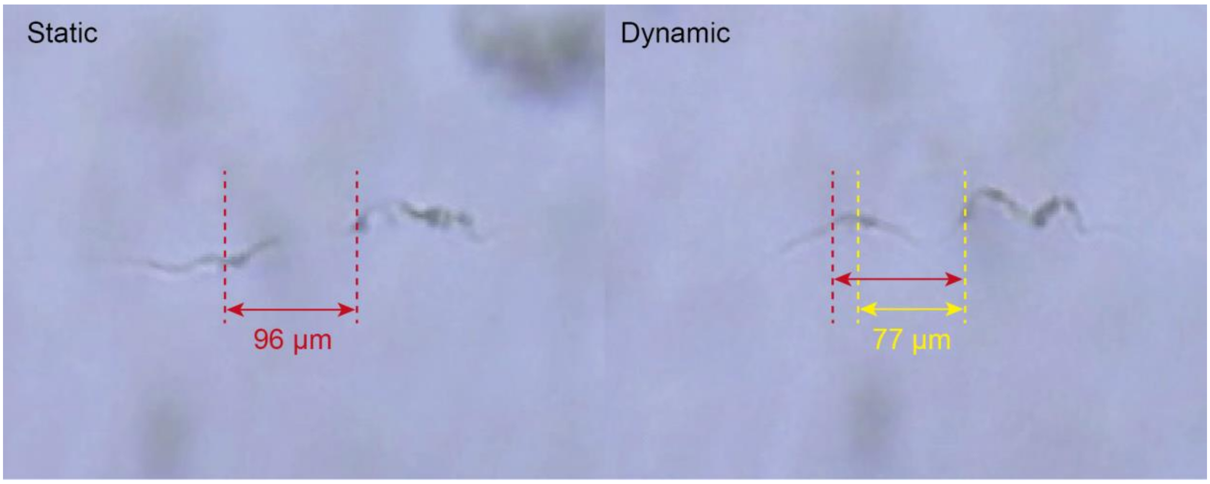

Supplementary Figure 8 | Shapes of the slender tail under static and dynamic conditions. The experimental results suggest that flexible tail forms into helical shape under dynamic condition. The behavior is nuanced: the rigid, less deformable helical head predominantly exhibits helical propulsion when actuated by the external magnetic field. However, in the slender tail section, the rotation of the head induces the generation of helical tail. This coexistence is substantiated by a comparison of the dimensions and shapes of the slender tail under static and dynamic conditions. Compared with the static condition, helical shape is observed at the tail of the microrobot and the length is shortened by 19%, which proves that the tail deforms in the dynamic state rather than simply helical propulsion of head.

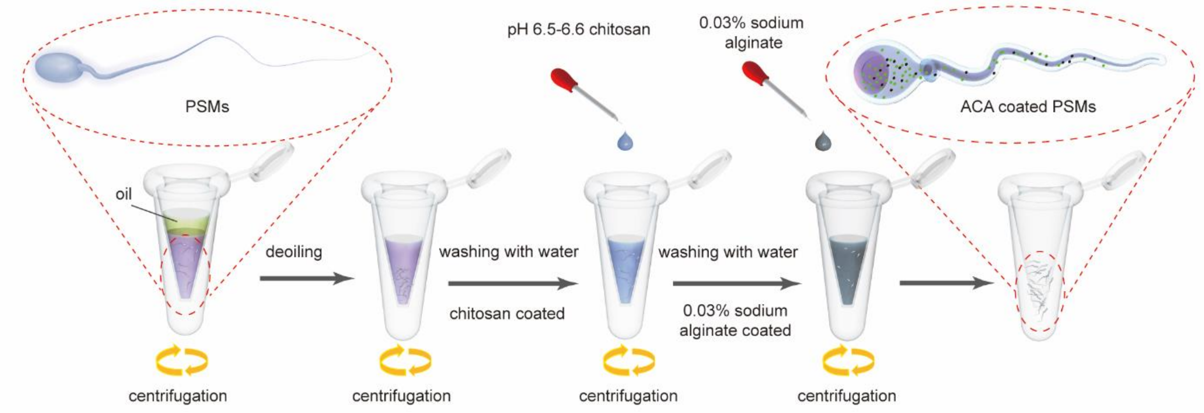

Supplementary Figure 9 | The process of ACA coating on the surface of PSMs. Through stepwise coating chitosan (pH 6.5-6.6) and light concentration sodium alginate (0.03 w/v%) layers on the surface of the sperm-liked microswimmer. During this process, the step washing is the key on keeping the package success rate. These methods are aimed at minimizing aggregation and preserving the individual integrity of the microswimmers throughout the experimental processes.

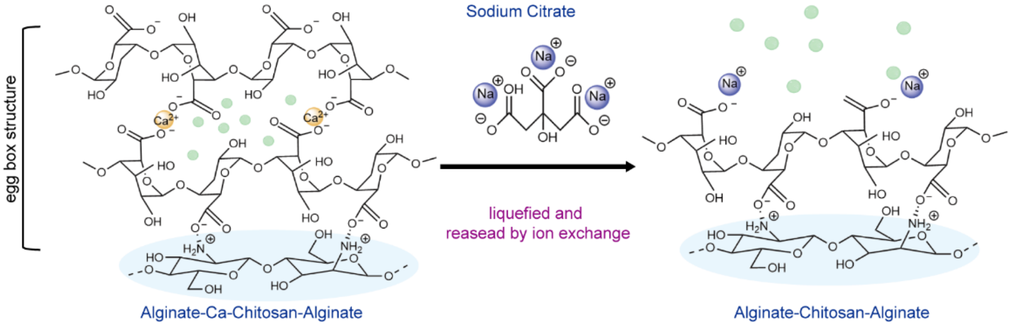

Supplementary Figure 10 | The release mechanism of ACA coated PSMs. The liquefication reaction is result of an intimate interaction between the carboxylic groups in sodium citrate and Ca^{2+} ions. With the ion exchange reaction between Ca^{2+} ions in Ca-alginate and Na^{+} ions in sodium citrate, drug nanoparticle is released through the ACA membrane.

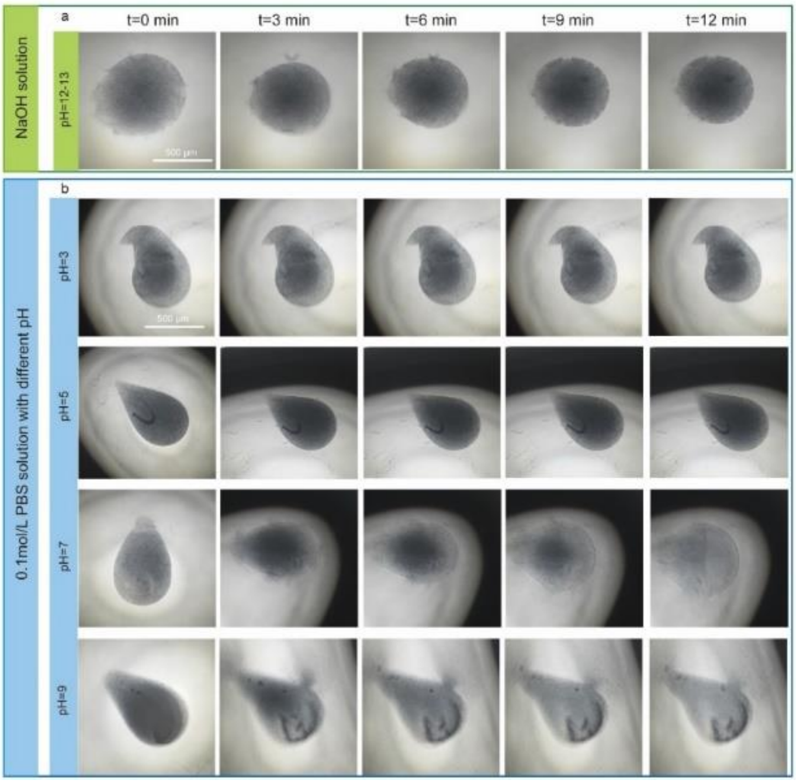

Supplementary Figure 11 | a, high pH (0.6M sodium hydroxide solution (pH=12-13)) without chelation. b, chelation with different pH (100µl of the same concentration PBS with different pH value (0.1mol L^-1, pH=3, 5,7, 9, Yuanye company, Shanghai). The results indicate that liquefaction cannot proceed without either ion chelation or weakly acidic to alkaline pH.

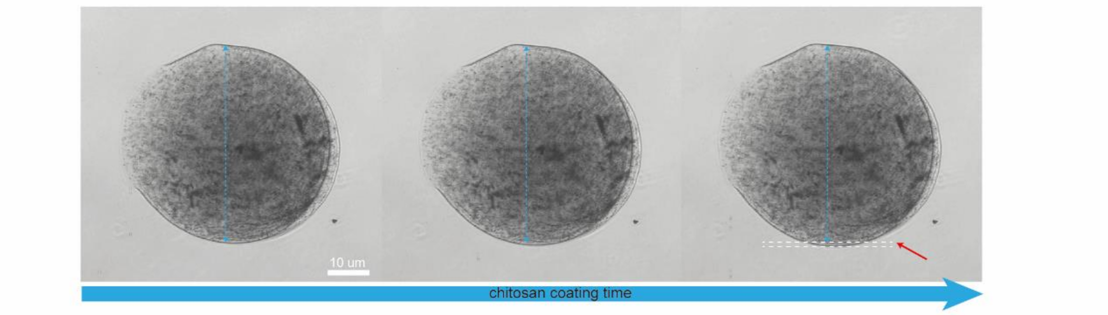

Supplementary Figure 12 | The thickness of the spherical microrobot increases with the duration of chitosan coating.

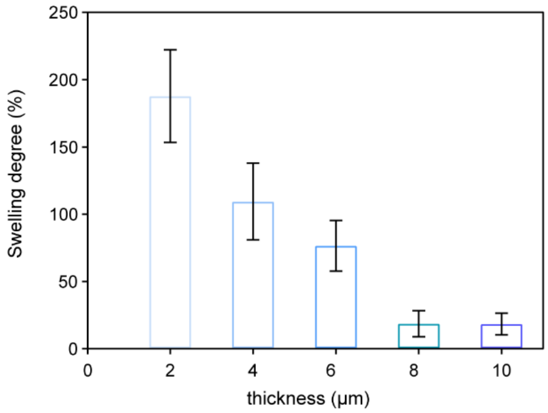

Supplementary Figure 13 | The swelling behavior of ACA coated PSMs. The result indicated that strength of ACA PSMs decreased with thinning membrane. Error bars indicate the standard deviation for n = 3 measurements at each data point.

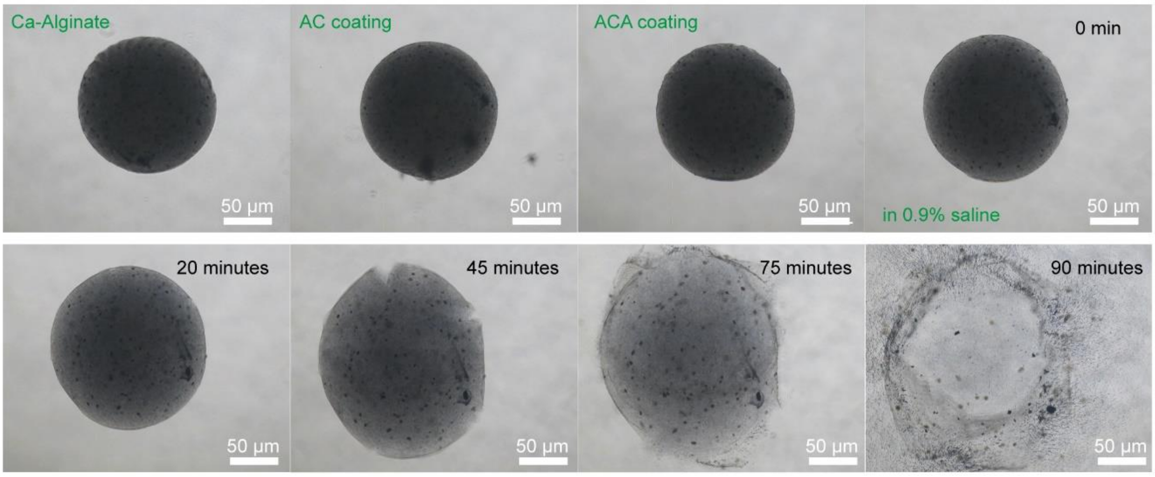

Supplementary Figure 14 | The durability of alginate-based microswimmers in 0.9% sodium chloride saline solution (pH~5.5).

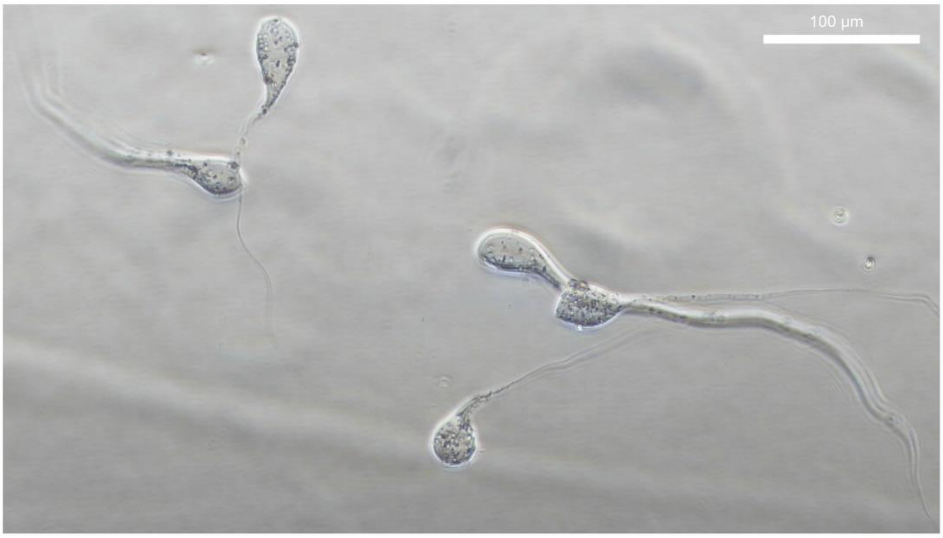

Supplementary Figure 15 | The distribution of iron oxide particles in the microswimmers.

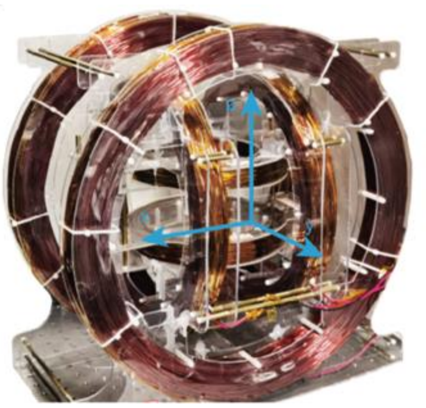

Supplementary Figure 16 | Helmholtz coils are used to generate a uniform magnetic field. The magnetic control system comprises three sets of coils, each with distinct sizes. Specifically, the coil radii are 40 cm, 22.5 cm, and 15 cm, respectively. Each pair of coils consists of two identical coils positioned at a separation distance equal to their individual radii.

Supplementary Tables

Table S1. The parameters for numerical calculations of propulsive force

Parameters		Microswimmer with regular head	Microswimmer with irregular head	Microswimmer with helix head
Head dimension	Helix angle (β_h)	90°	90°	47.5°
	Helix radius (R_h)	0 μm	0 μm	5.2 μm
	Equivalent body width (D_h)	12.7 μm	6.9 μm	3.7 μm
	Cross-sectional radius (b_h)	6.3 μm	3.4 μm	1.8 μm
	Helix length (L_h)	18.1 μm	27.3 μm	36.6 μm
Tail dimension	Straightened length (S_t)	82.5 μm	69.1 μm	72.3 μm
	Helix length (L_t)	69.6 μm	59.4 μm	62.1 μm
	Minimum cross-sectional width (D_{t0})	1.8 μm	1.5 μm	1.6 μm
	Maximum cross-sectional width (D_t)	5.9 μm	4.9 μm	3.7 μm
	Minimum cross-sectional radius (b_{t0})	0.9 μm	0.7 μm	0.8 μm
	Maximum cross-sectional radius (b_t)	2.9 μm	2.4 μm	1.8 μm
	Maximum helix radius (R_t)	9.1 μm	14.7 μm	15.1 μm
Magnetic field	Magnetic strength (H)	10 mT	10 mT	10 mT
	Tilt angle (θ)	30°	30°	30°
	Rotation frequency (f)	2 Hz	2 Hz	2 Hz
Viscosity		8.90 ×10^-4 Pa·s	8.90 ×10^-4 Pa·s	8.90 ×10^-4 Pa·s

Table S2. The comparison of sperm-like microswimmers

Author	Fabrication methods	Robot size	Morphology	Propulsion methods	Ref
Ming You, et. al	Magnetic assembly + in-situ polymerization	30 μm	R-head	BW	35
Veronika Magdanz, et. al	Electrostatic self-assembly	34 μm	R-head	HP	36
Friedrich Striggow, et. al	Biological template + Lithography	70 μm	R-I-head	BP	37
Haifeng Xu, et. al	Biological template + Lithography	80 μm	I-head	BW	38
Mariana Medina-Sanchez, et. al	Biological template	50 μm	R-head	HP	39
Islam S. M. Khalil, et. al	Lithography	322 μm	R-head	BW	40
Veronika Magdanz, et. al	Biological template	85 μm	I-head	BP	41
This work	Microfluidic chip	80-120 μm	R-H-I-head	DHP	N.A.

*R-head means regular head, I-head means irregular head, H-head means helical head;

**BW means beating waves, BP means biological propulsion, HP means helical propulsion, DHP means dual-helical propulsion.

Table S3. The COMSOL material parameters and physic field boundaries conditions

density of alginate, oil, $CaCl_2$ mixture	1.6×10^3 kg m^-3, 800 kg m^-3 and 1000~1025 kg m^-3, respectively (see Methods)
viscosity of alginate, oil, $CaCl_2$ mixture	0.0137 Pa.s, 0.032 Pa.s, and 0.00325 Pa.s, respectively (see Methods)
inlet1 velocity	0.6-1 m s^-1, measured by experimental value[47]
inlet2 velocity	0.0212-0.0255 m s^-1, measured by experimental value
outlet pressure	0 Pa
alginate phase, defined by ($\phi_{alginate}+\phi_{oil}+\phi_{calcium}=1$)	$\phi_{alginate}=1$, $\phi_{oil}=0$, $\phi_{calcium}=0$
oil phase, defined by ($\phi_{alginate}+\phi_{oil}+\phi_{calcium}=1$)	$\phi_{alginate}=0$, $\phi_{oil}=1$, $\phi_{calcium}=0$
calcium solution phase, defined by ($\phi_{alginate}+\phi_{oil}+\phi_{calcium}=1$)	$\phi_{alginate}=0$, $\phi_{oil}=0$, $\phi_{calcium}=1$, as the boundaries condition of inlet
alginate droplet concentration	*c*=740.33mol m^-3 (M_{alg}=216.121g mol^-1), calculated by experimental value
the inlet in "Turbulent Flow"	*c*=0

Supplementary Notes

Note S1- Droplet formation

As the microfluidic cross-junction shown in Fig. 2a, where the alginate-oil droplets form via three inlets, i.e., one for the dispersed flow phase (alginate) and two others for continuous flow phases (oil). Normally, according to the low Reynolds number in such microfluidic chip, the fluid flows are laminar. Therefore, the Navier-Stokes and Cahn-Hilliard equations can be utilized to describe the hydrodynamics of the fluid flows[23,24]:

$$\begin{aligned} &\nabla \cdot \mathbf{u} = 0 \\ &\rho\left(\partial_t \mathbf{u} + (\mathbf{u}\cdot\nabla)\mathbf{u}\right) = -\nabla \cdot p\mathbf{I} + \nabla \cdot \left(\mu\left(\nabla\mathbf{u} + (\nabla\mathbf{u})^{\mathrm{T}}\right)\right) \\ &\partial_t \alpha + \mathbf{u}\cdot\nabla\alpha = \nabla\cdot\left(M\nabla\eta\right) \end{aligned} \tag{1}$$

where $\mathbf{u}$ is the fluid velocity, ρ is the fluid density, μ is the dynamic viscosity, α is the normalized density difference of two fluid flows, M is the Cahn-Hilliard mobility, and η is the chemical potential. p and $\mathbf{I}$ are the fluid static pressure and unit tensor respectively, which can be derived through the Gibbs-Duhem relation[42]:

$$\nabla \cdot p\mathbf{I} = \nabla\left(\rho {c_s}^2\right) + \alpha\nabla\eta \tag{2}$$

where c_s is the lattice speed of sound. Considering the difficulty for evaluation of $\nabla\eta$ when take the fluid-solid interaction into account, such equation can be reformed as:

$$\nabla \cdot p\mathbf{I} = \nabla p - \eta\nabla\alpha \tag{3}$$

where $p = \rho {c_s}^2 + \alpha\eta$ is the modified pressure. Considering the incompressible two-phase system (alginate-oil) with fluid densities ρ_h and ρ_o, through the Ginzburg-landau free energy function, the thermodynamics can be represented as:

$$F\left(\rho,\alpha,\nabla\alpha\right)=\int\left(\Psi\left(\alpha\right)+\frac{1}{2}\kappa\left|\nabla\alpha\right|^{2}+\rho c_{s}{}^{2}\ln\rho\right)dV \tag{4}$$

where the total density $\rho=\rho_h+\rho_o$, the density difference $\alpha=\left(\rho_h-\rho_o\right)/\rho$, the bulk free energy density $\Psi\left(\alpha\right)=\left(a\left(\alpha^2-1\right)^2\right)/4$ with positive constant a, κ relates to the interfacial tension σ and $\left(\kappa\left|\nabla\alpha\right|^2\right)/2$ represents the interface energy. Based on Equation 3, the chemical potential η can be calculated as:

$$\eta=\frac{\delta F\left(\rho,\alpha,\nabla\alpha\right)}{\delta\alpha}=\Psi'\left(\alpha\right)-\kappa\nabla^2\alpha=a\alpha\left(\alpha^2-1\right)-\kappa\nabla^2\alpha \tag{5}$$

Define the thickness of alginate-oil interface λ, which can be defined as:

$$\lambda=b\sqrt{\frac{2\kappa}{a}} \tag{6}$$

where b is the constant coefficient. Define the spatial location normal to the interface z, the thickness of alginate-oil interface λ can be represented as:

$$\lambda=\frac{2bz}{\ln\left(1+\alpha\left(z\right)\right)-\ln\left(1-\alpha\left(z\right)\right)} \tag{7}$$

Assuming the interfacial tension in equilibrium on the plane interface, which can be calculated through[43]:

$$\sigma=\int_{-\infty}^{+\infty}\kappa\left(\frac{d\alpha}{dz}\right)^2 dz \tag{8}$$

Therefore, the thickness of alginate-oil interface λ can be obtained:

$$\lambda = \frac{4b\kappa}{3\sigma} \tag{9}$$

The Navier-Stokes and Cahn-Hilliard Equation 1 for alginate-oil droplet formation dynamic description can be solved through lattice Boltzmann scheme[44,45]:

$$f_i^k\left(\mathbf{x}+\mathbf{e}_i, t+1\right) - f_i^k\left(\mathbf{x}, t\right) = \Theta_i^k\left(\mathbf{x}, t\right) \tag{10}$$

where $f_i^k\left(\mathbf{x}, t\right)$ represents the particle distribution at position $\mathbf{x}$ and time t for the fluid with ith velocity direction. $\mathbf{e}_i$ represents the particle velocity in ith velocity direction, and $\Theta_i^k\left(\mathbf{x}, t\right)$ represents the collision operator.

Note S2- Demulsification

During the demulsification, the fabricated alginate-oil droplet is imported to the vortex flow generated via magnetic stirrer in a beaker (Supplementary Fig. 2). And then, the droplet exerts large deformation and demulsificates in the vortex flow. In this process, the vortex flow need to be characterized in the first place. Through the applied magnetic field with adjustable frequency, the magnetic stirrer is controlled to rotate and generate strong stirring fluid motion. The vortex flow is modelling in cylindrical velocity components, i.e., radial velocity (v_{vr}), tangential velocity (v_{vt}), and axial velocity (v_{vz}), which can be expressed as Burgers vortex[46]:

$$v_{vr} = -\frac{2\nu_v}{l_v^{\ 2}} r_v$$

$$v_{vt} = \frac{C_v}{r_v}\left(1 - e^{-\frac{r_v^{\ 2}}{c_v^{\ 2}}}\right)$$

$$v_{vz} = \frac{4\nu_v}{c_v^{\ 2}} z_v \tag{11}$$

where ν_v represents the fluid viscosity in the beaker. The vorticity is assumed to distribute in a cylinder (central line z_v) with radius c_v, and C_v is circulation constant. The vortex strength C_v can be calculated through:

$$C_v = f_v i_v^{\ 2}\left(\frac{\beta_v^{\ 2}\nu_v}{\ln 2\left(\alpha_v H_v + \gamma_v R_v\right)\left(gR_v\right)^{\frac{1}{2}}}\right)^{\frac{1}{2}} \tag{12}$$

where f_v is the vortex frequency, g is the gravitational acceleration. α_v, β_v, and γ_v are fitting parameters can be obtained in reference[47]. As shown in Supplementary Fig. 1, i_v, j_v, k_v, H_v, h, and R_v represent the length of the stirrer, the halfwidth of the vortex, the height of the stirrer, the height of still solution, the distance between the vortex's deepest point and the bottom of the beaker, the radius of the beaker. The radius of the cylinder c_v can be obtained through:

$$c_v = \frac{\beta_v i_v \nu_v}{k_v\left(gR_v\right)^{\frac{1}{2}}} \tag{13}$$

Therefore, radial velocity, tangential velocity, and axial velocity of the vortex can be investigated. After that, the droplet mechanics need to be determined during the alginate-oil capsule large deformation and burst process. Consider the fabricated alginate-oil droplet with unstressed geometry in the initial state. We assume that the oil membrane is isotropic and hyperelastic with surface elastic modulus E_o, which follows the neo-Hookean constitutive law. According to previous analytical researches, the deformation of such spherical droplet mainly depends on two parameters, i.e., capillary number Ca and viscosity ratio of the interior droplet to exterior λ_d. In the steady state, when the flow strength increase, the droplet remains unburst until a critical capillary value Ω_b reached, which can be expressed as[48-51]:

$$Ca = \frac{\mu_d v_{ve}}{E_o d_d}, \quad \begin{matrix} Ca < \Omega_b & \text{unburst} \\ Ca \geq \Omega_b & \text{burst} \end{matrix} \tag{14}$$

where μ_d is the dynamic viscosity of oil membrane, d_d is the droplet diameter. Therefore, the shear velocity v_{ve} is the critical parameter that influence the demulsification process. Consider the thickness of alginate-oil interface λ is much small that can be negligible, the droplet deformation gradient $\mathbf{F}_d$ can be given as:

$$\mathbf{F}_d\left(\mathbf{X}_d, t_d\right) = \frac{\partial \mathbf{x}_d\left(\mathbf{X}_d, t_d\right)}{\partial \mathbf{X}_d} \tag{15}$$

where $\mathbf{X}_d$ is a material point of the unstressed state, and $\mathbf{x}_d$ is the position vector after deformation. According to the neo-Hookean constitutive law related to the strain energy function w_d, the surface elastic modulus E_o can be expressed as:

$$E_o = \frac{2w_d}{\chi_1^2 + \chi_2^2 - 3 + \left(\chi_1^2 \chi_2^2\right)^{-1}} \tag{16}$$

where χ_1 and χ_2 are the principle extension ratios. The Cauchy stress tensor $\mathbf{T}_d$ can be calculated as:

$$\mathbf{T}_d = \frac{1}{\chi_1 \chi_2} \mathbf{F}_d \cdot \frac{\partial w_d}{\partial \mathbf{e}_d} \cdot \mathbf{F}_d^{\mathrm{T}} \tag{17}$$

where $\mathbf{e}_d$ is the Green-Lagrange strain tensor, define the tangential projection operator $\mathbf{I}_d$, $\mathbf{e}_d$ can be represented as:

$$\mathbf{e}_d = \frac{1}{2}\left(\mathbf{F}_d^{\mathrm{T}} \cdot \mathbf{F}_d - \mathbf{I}_d\right) \tag{18}$$

Note S3- The description of COMSOL model setup

The mechanism of drop deformation is regulated by the value of the capillary number, Ca .The critical value of the capillary number at which the states transits from the stable to the deformation states is found to be approximately$\sim$ 0.015 [52-54]. In this case, the Ω_b $\sim$0.0032-0.0128. The result presents $\Omega_b < Ca$, which reasonably close to the theoretical in the experiments.

In our model 50 μm sphere shaped alginate droplet (with 0.5 μm oil thickness) is dropped into a 0.3 mm width and 3 mm length of vortex liquid channel (calcium chloride solution). The aim of the simulation is to predict the demulsification process of hydrogel-oil droplets in a transient manner. The geometry of the experimental set-up is shown in Supplementary Fig. 4. The motions of the hydrogel-oil droplets, injected from the upper-left inlet, are simulated and plotted in 2D planar simulation.

Under the effect of gravity, hydrogel droplets with a density higher than that of the oil fluid flow along in the channel as they simultaneously flow to the right together with the oil flow. A clear shaping can be captured on the oil–water interface, and the oil film can break after vortex

flow impact. The Supplementary Fig. 5 shows the droplets are in a turbulent fluid and are being impacted from all directions. The demulsification are shown in Video S1.

For the geometric parameters, the droplet diameter, oil thickness, and inner diameter of the glass tube is set as 50 μm, 0.5 μm, and 100 μm, respectively. These parameters are set based on the measurements in the experiments. The detailed material parameters and physic field boundaries conditions are given in Supplementary Table S3.

These simulations are performed in 2D planar, which aimed to study 1: the demulsification process of the hydrogel-oil droplets 2: the formation process of the microswimmer (alginate droplet concentration diffusion process). We utilize COMSOL Multiphysics to simulate these two processes. Generally, we use "Turbulent Flow" to simulate the flow, and set up two inlet conditions to represent the flow in the bottle (Supplementary Fig. 4, inlet1) and the flow induced by alginate dropping into the calcium mixture solution (Supplementary Fig. 4, inlet2). The velocity are 0.6-1 m s^-1 and 0.0212-0.0255 m s^-1, which are based on experimental values[47]. The inserted capillary glass tube is defined as the "interior wall", while all walls in the flow model arte assumed to have a no slip "wall condition", which consistent with the experimental situation. The outlet condition (Supplementary Fig. 4, outlet) is set with an static pressure '0 Pa'.

To simulate the demulsification process of the hydrogel-oil droplets, we use "Ternary Phase Field" to couple with the "Turbulent Flow", while setting up "Three Phase Flow, Phase Field" in multiphysics. In this three-phase flow field, we can simulate flow of three immiscible fluids separated by moving interface. The phase field variables vary between 0 and 1 and are a measure of the concentration of each phase. At each point, the phase field variables satisfy the following equation:

$$\phi_{a\lg inate} + \phi_{oil} + \phi_{calcium} = 1 \tag{19}$$

Based on the above definition, we set boundary conditions according to three materials. The boundary conditions of alginate phase is $\phi_{a\lg inate} = 1$, $\phi_{oil} = 0$, $\phi_{calcium} = 0$, oil phase is $\phi_{a\lg inate} = 0$, $\phi_{oil} = 1$, $\phi_{calcium} = 0$ and the $CaCl_2$ mixture is $\phi_{a\lg inate} = 0$, $\phi_{oil} = 0$, $\phi_{calcium} = 1$. We choose the boundaries which is set the inlet in "Turbulent Flow" as inlet of "Ternary Phase Field". The boundaries condition

of inlet is $\phi_{alginate}=0$, $\phi_{oil}=0$, $\phi_{calcium}=1$. After setting these boundaries, we finally get the demulsification process of the hydrogel-oil droplets, as shown in Maintext Fig. 2b and Supplementary Movie 1.

To simulate the formation process of the microswimmer (alginate droplet concentration diffusion process), we use "Transport of Diluted Species" to couple with the "Turbulent Flow", while setting up "Reacting Flow, Diluted Species" in multiphysics. In transport of diluted species field, we set the initial concentration of alginate droplet, the concentration boundaries was *c*=740.33mol m^-3 (Malg=216.121g mol^-1), which are based on experimental values. We set the condition *c*=0 on the boundary which is set the inlet in "Turbulent Flow", meaning the inflowing liquid is $CaCl_2$ mixture. The use of "Transport of Diluted Species" is to characterize the alginate-oil shape, because there is a short concentration diffusion process between demulsification and gelation, since the concentration contours can be used to characterize the microswimmer's deformation[55,56].

Note S4- Solidification

When the alginate-oil droplets burst in the vortex flow, the inside alginate phase will outflow and solidify in the calcium chloride solution via cross-linking reaction. As modeled in Equation 11, the alginate droplets suffer radial velocity (v_{vr}), tangential velocity (v_{vt}), and axial velocity (v_{vz}) and exert deformation in the vortex flow. During the extension formation of the alginate, several parameters are determined, i.e., capillary number Ca_s, Reynolds number Re_s, and viscosity ratio λ_s:

$$Ca_s = \frac{r_s \dot{\gamma}_s \mu_s}{\sigma_s},\ Re_s = \frac{\rho_s \dot{\gamma}_s {r_s}^2}{\mu_s},\ \lambda_s = \frac{\mu_h}{\mu_s} \tag{20}$$

where r_s, $\dot{\gamma}_s$, μ_s, σ_s, ρ_s, and μ_h represent the initial radius of alginate droplet, the average shear rate, the viscosity of the calcium chloride solution, the interfacial tension, the fluid density, and the viscosity of the alginate droplet. The governing equations are[57]:

$$\nabla \cdot \mathbf{u}_s = 0$$
$$\rho_s \left(\partial_t \mathbf{u}_s + (\mathbf{u}_s \cdot \nabla) \mathbf{u}_s \right) = -\nabla \cdot p_s \mathbf{I}_s + \nabla \cdot \tau_s + \mathbf{F}_s \tag{21}$$

where $\mathbf{F}_s$ and τ_s represents the interfacial tension force and viscous stress. Based on the Bingham equation, τ_s can be calculated as:

$$\tau_s = \dot{\gamma}_s \mu_p + \frac{\dot{\gamma}_s \tau_y}{|\dot{\gamma}_s|} \left(1 - \exp\left(-m_s |\dot{\gamma}_s|\right)\right) \tag{22}$$

where μ_p, τ_y and m_s are the constant plastic viscosity, the apparent yield stress and the stress growth exponent respectively. After the extension of the alginate droplet, the structure formation Ω_s depends on interfacial tension σ_s, the flow acceleration at the demulsification position a_s, extension volumetric rate Q_s with tip diameter d_s, fluid density ρ_s, and kinematic viscosity μ_h. Based on Buckingham's Π-theorem, several dimensionless parameters are defined as[25,58]:

$$\Pi_1 = \left(\frac{\mu_h{}^5}{a_s Q_s{}^3} \right)^{\frac{1}{5}},\ \Pi_2 = \left(\frac{\mu_h Q_s}{a_s d_s{}^4} \right)^{\frac{1}{4}},\ \Pi_3 = \left(\frac{\sigma_s d_s{}^4}{\rho_s \mu_h Q_s} \right) \tag{23}$$

Therefore, the dimensionless formed structure Π_{Ω_s} obeys a law of the form:

$$\Pi_{\Omega_s} = fct\left(\Pi_1, \Pi_2, \Pi_3\right) \tag{24}$$

Considering the different demulsification positions with various flow acceration values in the vortex flow, the helix, regular, irregular head polymorphous microswimmers can be fabricated.

Note S5- The explanation of physical principles and simulation

The tail formation of the microswimmer can be divided into three processes, i..e, (1) vortex flow generation, (2) droplet deformation, and (3) demulsification. To elucidate the principle and find out the key factors determining these processes, we conduct research through theoretical analysis, simulations, and experiments, where the simulation is crucial. Briefly: The purpose and significance of the simulations in each step are explained below:

(1) Vortex flow generation: We have established a mathematic model to investigate this process, which suggests that the shear velocity is the critical parameter (Eq S11). Yet, it's difficult to calculate the shear velocity at the demulsification position due to the complex parameters in the model. Therefore, we conduct simulation to investigate the range of the shear velocity. Our simulation suggests that when the rotation speed of magnetic stirrer increases from 800-1000 rpm, the shear velocity increases from 0.6 m s^-1 to 1 m s^-1 at the demulsification position in the vortex flow. These values are important for the following droplet deformation modeling and simulation analysis.

(2) Droplet deformation: In this process, one key challenge is to figure out the droplet deformation in the vortex flow and the droplet burst state, which is also hard to obtain analytical solutions through modeling calculations (Eq S14). Therefore, we further delved into the mechanics of the droplet during the large deformation and burst process of the alginate-oil capsule through the simulation. Our simulation suggests that when the shear velocity reach the 0.6 m s^-1 (corresponding to magnetic stirrer rotation speeds of 800rpm), the droplet would burst and generate a tiny tail. These values are important for the following demulsification analysis.

(3) Demulsification: After obtaining the trigger value of shear velocity that can generate the microswimmer with tail structure, we need figure out the polymorphous-tailed microswimmer generation progress, where the transient demulsification process of hydrogel-oil droplets is the key challenge. Therefore we have simulated the demulsification process with a 50 μm diameter sphere-shaped alginate droplet (0.5 μm oil thickness), which allowed us to observe the transient demulsification process of hydrogel-oil droplets. We have observed the hydrogel droplets' behavior in tandem with the oil flow, capturing the shaping on the oil-water interface and the subsequent oil film breakage due to the impact of the vortex flow. These outcomes consistently aligned with our theoretical analyses and experimental observations.

The simulation environment we employ in this manuscript is 2D planar. The reason is that our goal is to investigate the influence of shear velocity on the fabrication process of microswimmers, where 2D planar simulation can take all key factors into consideration while maintaining a low time and computing resource consuming. The experimental observation showcases that the demulsification and deformation process is confined to micrometer size and milli-seconds, which is orders of magnitude smaller than the dimension of the simulation environment. Therefore, the shape of the simulated domain and the shape of the vial would not influence the results significantly.

Note S6- The dynamic model of sperm liked microswimmer

As shown in Supplementary Fig. 7, when a rotating magnetic field with a tilt angle θ about the locomotion direction OO’ is applied, the head of microswimmer will revolve around locomotion direction OO’ and rotate along its own helix axis AA’ simultaneously. Following the revolving of head, the slender tail forms into a spiral shape. According to the mechanical analysis, both the rigid head and flexible tail make the contribution during the sperm locomotion.

As shown in the general model, the propulsion of helical element with a unit length can be expressed as:

$$\mathrm{d}F_r = dF_n sin\beta - dF_t cos\beta \tag{25}$$

In which,

$$\mathrm{d}F_n = \tau_n V_n ds = \tau_n \omega R cos\beta \tag{26}$$

$$\mathrm{d}F_t = \tau_t V_t ds = \tau_t \omega R sin\beta \tag{27}$$

where β is the pitch angle of spiral, $\mathrm{d}F_n$ and $\mathrm{d}F_t$ are normal and tangential resistance force respectively during rotation which related to the helical radius R, rotation speed ω and coefficients resistance τ.

For the revolving of head, the helix head can be considered as a whole unit with a long axis L_h and equivalent body width D_h, where $\beta = \theta$, $R_{h'} = L_x sin\theta$, $P_{h'} = \frac{2\pi R_{h'}}{tan\beta}$. Since the ratio $\frac{L_h}{D_h} < 10$, we adopt the resistive force theory to calculate the coefficients resistance:

$$\tau_{t1} = \frac{2\pi\mu}{\ln\left(\frac{2P_{h'}}{D_h}\right) - 0.5} \tag{28}$$

$$\tau_{n1} = \frac{4\pi\mu}{\ln\left(\frac{2P_{h'}}{D_h}\right) + 0.5} \tag{29}$$

Where, τ_{t1} and τ_{n1} are tangential and normal coefficients resistance of helical head respectively, $\mu = 1.0$ mpa · s is the dynamic viscosity of water. Then, the propulsion of the revolving of head under rotation speed ω can be expressed by integrating along the axis length L_h:

$$|\mathbf{F_1}| = F_{r1} = \int_0^{L_h} \omega L_x sin\theta(\tau_{n1} - \tau_{t1})\, sin\theta cos\theta dx \tag{30}$$

For the rotating of helical head, it has a small helix radius R_h and pitch angle β_h. The helix pitch P_h can be calculated by

$$P_h = \frac{2\pi R_h}{tan\beta_h} \tag{31}$$

Since the ratio $\epsilon = \frac{l_h}{b_h} < 10$, where l_h is the half wavelength of helical head and b_h is the cross-sectional radius of the element, we adopt the resistive force theory to calculate the coefficients resistance:

$$\tau_{t2} = \frac{2\pi\mu}{\ln\left(\frac{2P_h}{b_h}\right) - 0.5} \tag{32}$$

$$\tau_{n2} = \frac{4\pi\mu}{\ln\left(\frac{2P_h}{b_h}\right) + 0.5} \tag{33}$$

Then, the propulsion of the helical head along its helix axis can be expressed by integrating:

$$\mathrm{F}_{r2} = \int_0^{S_h} \omega R_h(\tau_{n2} - \tau_{t2})\, sin\beta_h cos\beta_h ds \quad (34)$$

Where S_h is the straightened length of helical head and can be given by:

$$S_h = \frac{L_h}{cos\beta_h} \quad (35)$$

Considering the tilt angle θ between head and locomotion direction, then the contributed propulsion force from head rotation can be further delivered as:

$$|\mathbf{F_2}| = F_{r2}cos\theta = \omega \mathrm{R}_h L_h(\tau_{n2} - \tau_{t2}) sin\beta_h cos\theta \quad (36)$$

For the rotation of flexible tail, assuming the radius of amplitude is R_t and the cross-sectional radius of the element changes from b_{t0} to b_t evenly. If the original straightened length of the tail is S_t and the axial length of tail after forming spiral is L_t. Then we can get the cross-sectional radius and pitch angle of the arbitrarily point x in the tail:

$$b_{tx} = b_t - \frac{x}{S_t}(b_t - b_{t0}) \quad (37)$$

$$\beta_t = arccos\frac{L_t}{S_t} \quad (38)$$

Since the ratio $\epsilon = \frac{l_t}{b_{tx}} > 10$, where l_t is the half wavelength of helical tail and b_{tx} is the cross-sectional radius of the element at point x, we adopt the slender body theory to calculate the coefficients resistance:

$$\tau_{t3}(x) = \frac{4\pi\mu}{\ln\left(\frac{0.0324{P_{tx}}^2}{{b_{tx}}^2}\right) - 1} \quad (39)$$

$$\tau_{n3}(x) = \frac{8\pi\mu}{\ln\left(\frac{0.0324{P_{tx}}^2}{{b_{tx}}^2}\right) + 1} \quad (40)$$

In which,

$$P_{tx} = 2\pi R_t \tan\left(arccos\frac{L_t}{S_t}\right) \tag{41}$$

Then, the propulsion of the flexible tail can be expressed by integrating:

$$|\mathbf{F_3}| = F_{r3} = \int_0^{S_t} \omega R_t(\tau_{n3}(x) - \tau_{t3}(x)) sin\beta_t cos\beta_t dx \tag{42}$$

Hence, the total propulsion of the microswimmer can be expressed as:

$$|\mathbf{F}| = |\mathbf{F_1} + \mathbf{F_2} + \mathbf{F_3}| = F_{r1} + F_{r2}cos\theta + F_{r3} \tag{43}$$

Note S7- The numerical calculations of propulsion

As shown in table S1 are the parameters of different types of microswimmer for numerical calculations. The parameters, including total length, helix radius, and average pitch angle, are obtained through sample observations in both static and dynamic states. While the determination of the helical shape of the flexible tail under rotation is intricately fitted through mathematical simulations, harmonizing with other observed parameters. Besides, the controllable actuation frequency and the liquid viscosity add a dynamic dimension that can be easily adjusted.

Since the axisymmetric of the regular head and the equivalent helix angle is 90°, the propulsion force from head is equal to the propulsion from the revolving of head, which can be calculated as:

$$F_{head1} = \int_0^{L_h} \omega L_x sin\theta(\tau_{n1} - \tau_{t1})\, sin\theta cos\theta dx = 4.3 \times 10\hat{} - 4\ \mu N \tag{44}$$

And the propulsion of tail can be calculated as:

$$F_{tail1} = \int_0^{S_t} \omega R_t(\tau_{n3}(x) - \tau_{t3}(x)) sin\beta_t cos\beta_t dx = 41.8 \times 10\hat{} - 4\ \mu N \tag{45}$$

Therefore, the total propulsion of the microswimmer with regular head can be expressed as:

$$F_{regular} = F_{head1} + F_{tail1} = 46.1 \times 10^{\wedge} - 4\ \mu N \quad (46)$$

Where the propulsive force from regular head and flexible tail contributes 9.3% and 90.7% to their total propulsive force respectively.

Similar to the regular head, the rotation of irregular head also doesn't contribute to the propulsion and mainly come from its revolving. The propulsion force from irregular head can be calculated as:

$$F_{head2} = \int_0^{L_h} \omega L_x sin\theta(\tau_{n1} - \tau_{t1})\, sin\theta cos\theta ds = 9.5 \times 10^{\wedge} - 4\ \mu N \quad (47)$$

And the propulsion of tail can be calculated as:

$$F_{tail2} = \int_0^{S_t} \omega R_t(\tau_{n3}(x) - \tau_{t3}(x)) sin\beta_t cos\beta_t dx = 59.1 \times 10^{\wedge} - 4\ \mu N \quad (48)$$

Then the total propulsion of the microswimmer with irregular head can be expressed as:

$$F_{irregular} = F_{head2} + F_{tail2} = 68.6 \times 10^{\wedge} - 4\ \mu N \quad (49)$$

Where the propulsive force from irregular head and flexible tail contributes 13.8% and 86.2% to their total propulsive force respectively.

For the microswimmer with helix head, the propulsion force from head including the rotation and revolving, which can be calculated as:

$$F_{head3} = \int_0^{L_h} \omega L_x sin\theta(\tau_{n1} - \tau_{t1})\, sin\theta cos\theta ds + cos\theta \int_0^{S_h} \omega R_h(\tau_{n2} - \tau_{t2})\, sin\beta_h cos\beta_h ds$$

$$= 17.6 \times 10^{\wedge} - 4 + 15.9 \times 10^{\wedge} - 4$$

$$= 33.5 \times 10^{\wedge} - 4\ \mu N \quad (50)$$

And the propulsion of tail can be calculated as:

$$F_{tail3} = \int_0^{S_t} \omega R_t(\tau_{n3}(x) - \tau_{t3}(x)) sin\beta_t cos\beta_t dx = 63.8 \times 10\hat{} - 4\ \mu N \quad (51)$$

Therefore, the total propulsion of the microswimmer with helix head can be expressed as:

$$F_{helix} = F_{head3} + F_{tail3} = 97.3 \times 10\hat{} - 4\ \mu N \quad (52)$$

Where the propulsive force from irregular head and flexible tail contributes 34.4% and 65.6% to their total propulsive force respectively.

Note S8- The swelling behavior of ACA coated PSMs

'Swelling behavior' is an intrinsic property of hydrogels, where the PSMs enlarge due to solvent penetration into the void space between the polymeric chain network (ACA coating process). The PSMs enlarge which affects their locomotion under the magnetic field. Therefore, we need to analyze the relationship between the swelling degree and coating time to optimize membrane thickness, thereby reducing the locomotion obstruction at micro scale. In this experiment, we have obtained difference thickness PSMs by changing coating time, and used an optical microscope to measure the uncoated PSMs's head diameter and PSMs's head diameter respectively. According to our previous research[47], swelling degree (Sw) is expressed as follows:

$$Sw\ (\%) = \left[\left(\frac{\text{Diameter}_{ACA}}{\text{Diameter}_{PSMs}}\right)^3 - 1\right]100 \quad (53)$$

The volume swelling degree of the PSMs with different membrane thicknesses was quite different as below. The PSMs with <6 μm membrane thickness exhibit the greatest swelling degree of 187.76% among the five groups. The result indicated that strength of ACA PSMs decreased with thinning membrane (Supplementary Fig. 10).

We found that suitable thickness can perform preferable sustain release ability, as shown in Fig. 4e. However, if the thickness is too thick or thin, the release properties of the

microswimmer cannot be maintained, because too thin or too thick thickness will respectively result in not fully crosslinked between chitosan and sodium alginate amide bonds or the swelling reaction. For example, when thickness＜6 μm, the cumulative release value reaches approximately 88-95% within 2 hours presents burst releasing due to rupture of thinner membrane. On the other hand, when thickness≥6 μm, the cumulative release value increase and reach around 35-50% before 2 hours, indicating a thicker membrane enables effectively alleviating the burst release. Yet in the following several hours, the cumulative release of thickness 8 μm to 10 μm, their release presents continuously increase and reach around 88%. Comparatively, the curve of release with a thickness ~ 6 μm is relatively flat around 60%, and presents the sustainable release property. The membrane with ~ 6 μm thickness demonstrates sustainable release ability to maintain a constant drug concentration in a period.
