# Supplementary material for: One-step formation of polymorphous sperm-like microswimmers by vortex turbulence-assisted microfluidics
Source: Nat Commun. 2024 Jun 4;15:4761. doi: 10.1038/s41467-024-49043-0 (PMC11150408; doi:10.1038/s41467-024-49043-0)
Supplement: Supplementary file 3 — Description of Additional Supplementary Files [file 41467_2024_49043_MOESM3_ESM.docx]

**Description of Additional Supplementary Files**

**File name: Supplementary Movie 1**

**Description:** The COMSOL stimulation of PSMs demulsification process.

**File name: Supplementary Movie 2**

**Description:** The locomotion of sperm-like microswimmers under different magnetic field frequency~1.

**File name: Supplementary Movie 3**

**Description:** The locomotion comparison between polymorphous sperm-like microswimmers.

**File name: Supplementary Movie 4**

**Description:** The liquefied fluorescence release of with/without ACA structure microswimmers.

**File name: Supplementary Movie 5**

**Description:** The thickness of the spherical microrobot increases with the duration of chitosan coating.
